# Supplementary material for: Short contacts between chains enhancing luminescence quantum yields and carrier mobilities in conjugated copolymers
Source: Nat Commun. 2019 Jun 13;10:2614. doi: 10.1038/s41467-019-10277-y (PMC6565747; doi:10.1038/s41467-019-10277-y)
Supplement: Supplementary file 1 — Supplementary Information [file 41467_2019_10277_MOESM1_ESM.pdf]

Supplementary information:

Short contacts between chains enhancing luminescence quantum yields and carrier mobilities in conjugated copolymers

Tudor H. Thomas[†], David J. Harkin[†], Alexander J. Gillett, Vincent Lemaur[1], Mark Nikolka, Aditya Sadhanala, Johannes M. Richter, John Armitage, Hu Chen[2], Iain McCulloch[2], S. Matthew Menke, Yoann Olivier[1], David Beljonne[1], and Henning Sirringhaus[*]

Cavendish Laboratory, University of Cambridge, JJ Thomson Avenue, Cambridge, CB3 0HE, UK

[1]Université de Mons, Place du Parc 20, 7000 Mons, Belgium

[2]KAUST Solar Center (KSC), KAUST, Thuwal 23955-6900, Saudi Arabia

[†]These authors contributed equally to this work.

[*]Corresponding author: hs220@cam.ac.uk.

Supplementary Figures

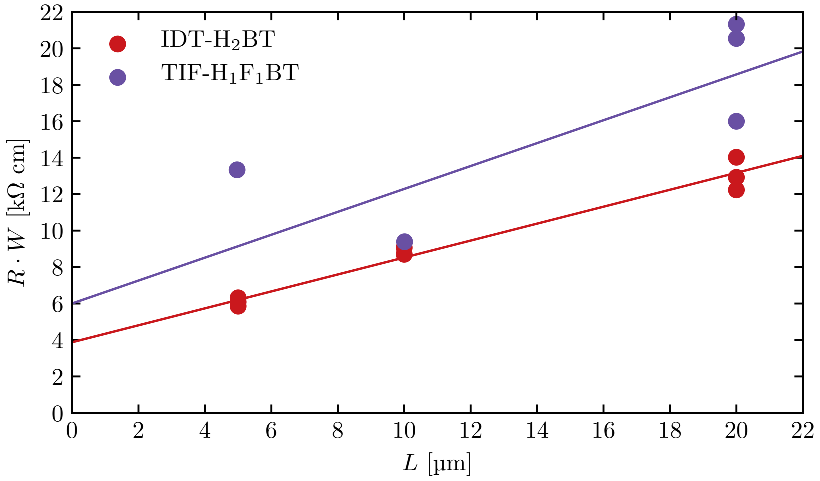

Supplementary Figure 1: Contact resistance of IDT-H_2BT and TIF-F_1H_1BT determined by the transfer length method.

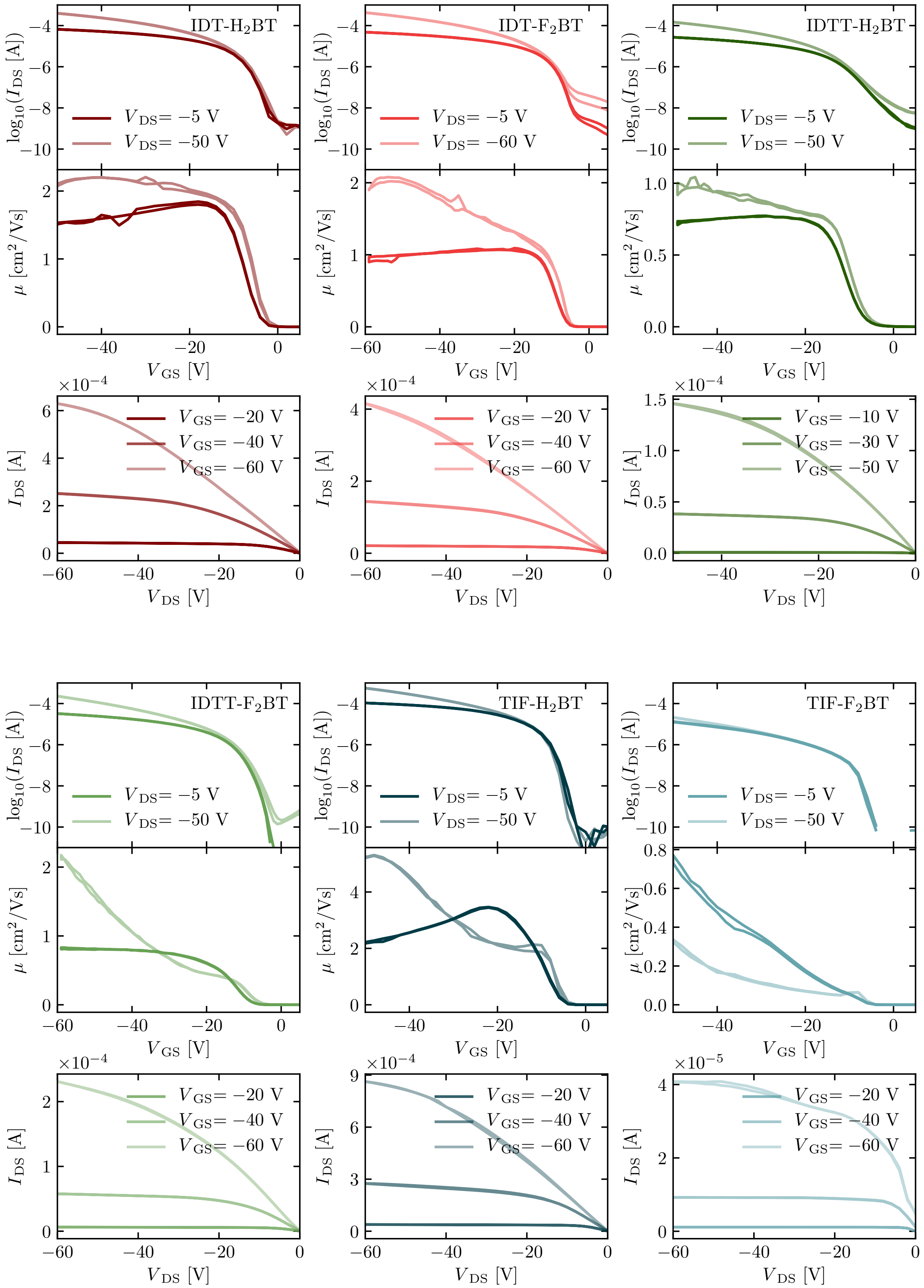

Supplementary Figure 2: Mobility extraction and OFET characteristics for polymers in this work.

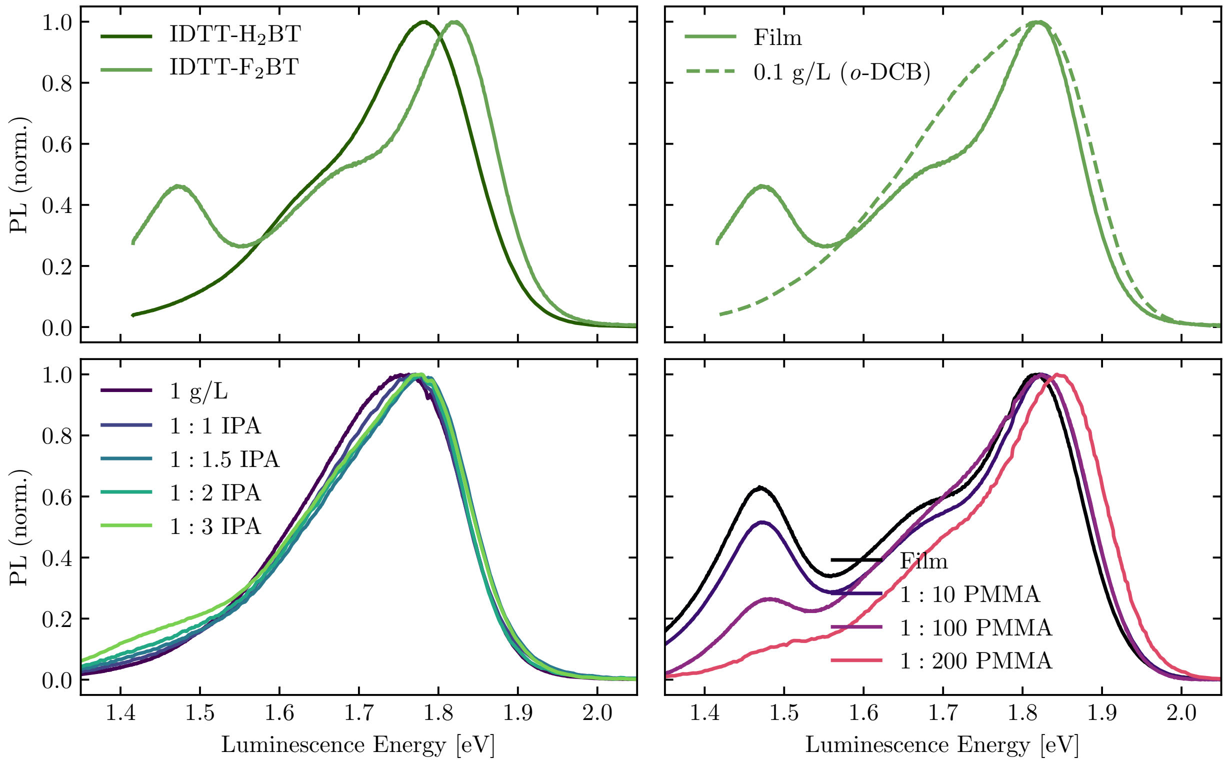

Supplementary Figure 3: Luminescent interchain species in IDTT-F_2BT: Thin film PL spectra of IDTT-H_2BT and IDTT-F_2BT (top left), IDTT-F_2BT in thin film and dilute solution (top right), solution spectra of IDTT-F_2BT with increasing volume ratio of non-solvent propan-2-ol (IPA) to introduce aggregation (bottom left), and IDTT-F_2BT blended with increasing weight fraction of poly(methyl methacrylate) (PMMA) (bottom right).

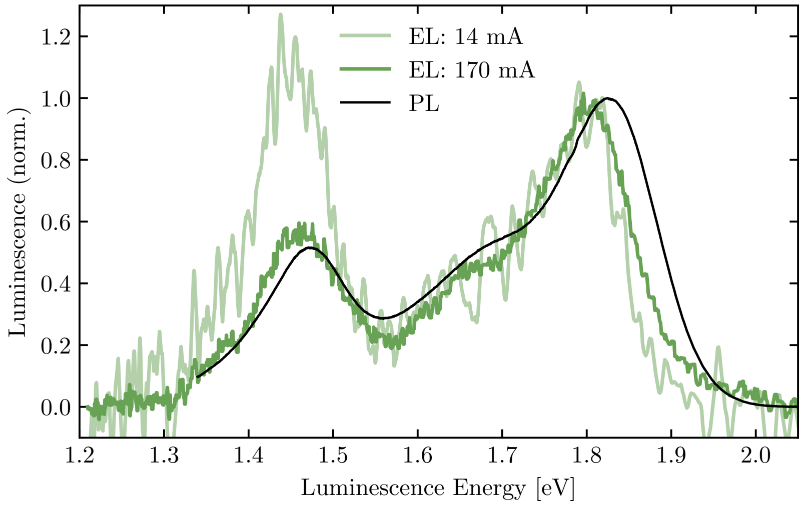

Supplementary Figure 4: Electroluminescence (and PL) of IDTT-F_2BT at different currents.

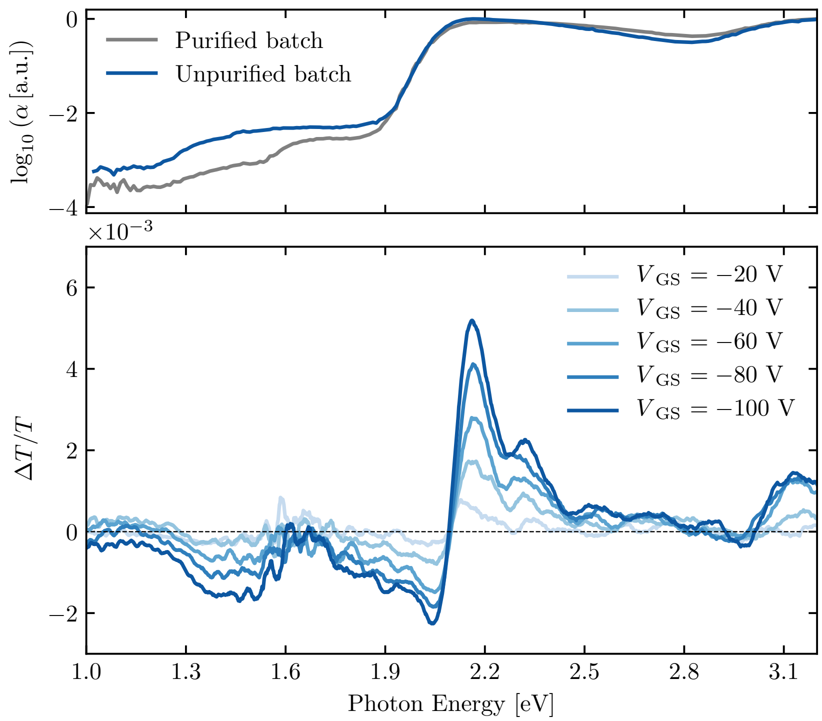

Supplementary Figure 5: Unintentional doping of TIF-H_2BT: Normalised PDS spectrum of purified and unpurified polymer (top) and CAS spectra at various gate biasing of a device fabricated from unpurified polymer.

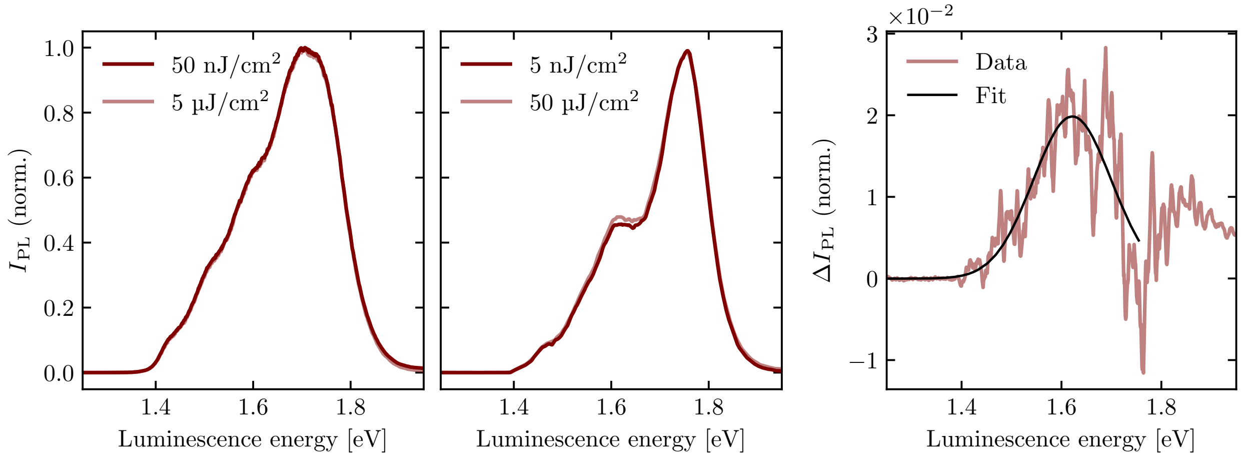

Supplementary Figure 6: PL spectra of IDT-H_2BT at different fluence: dilute solution (0.1g/L, *o*-DCB) (left) and thin film (centre) normalised to the maximum. Difference spectrum (right) of thin film PL at different fluence with Gaussian fit.

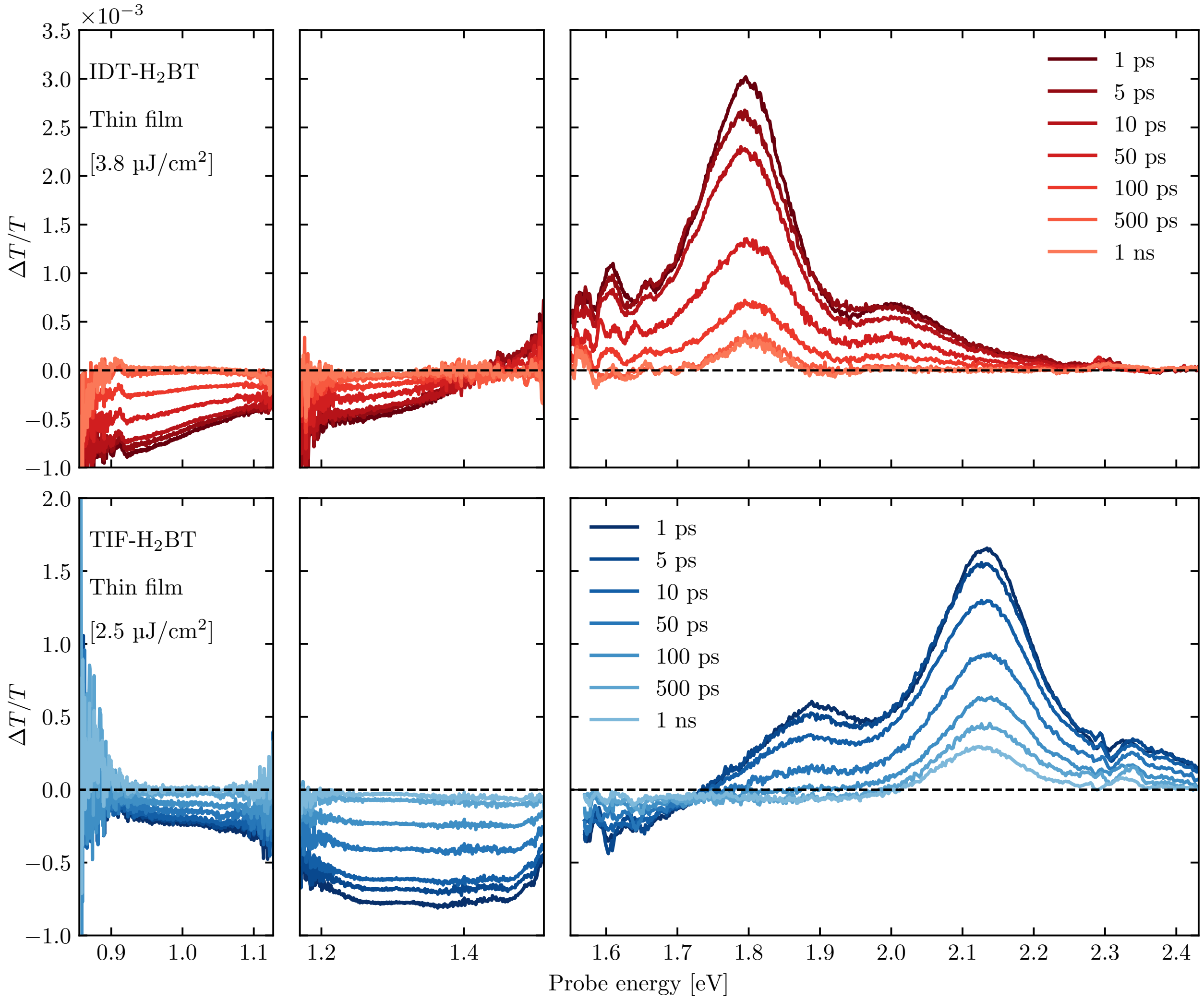

Supplementary Figure 7: TA spectral evolution from 1 ps−1 ns for IDT-H_2BT (top) and TIF-H_2BT (bottom).

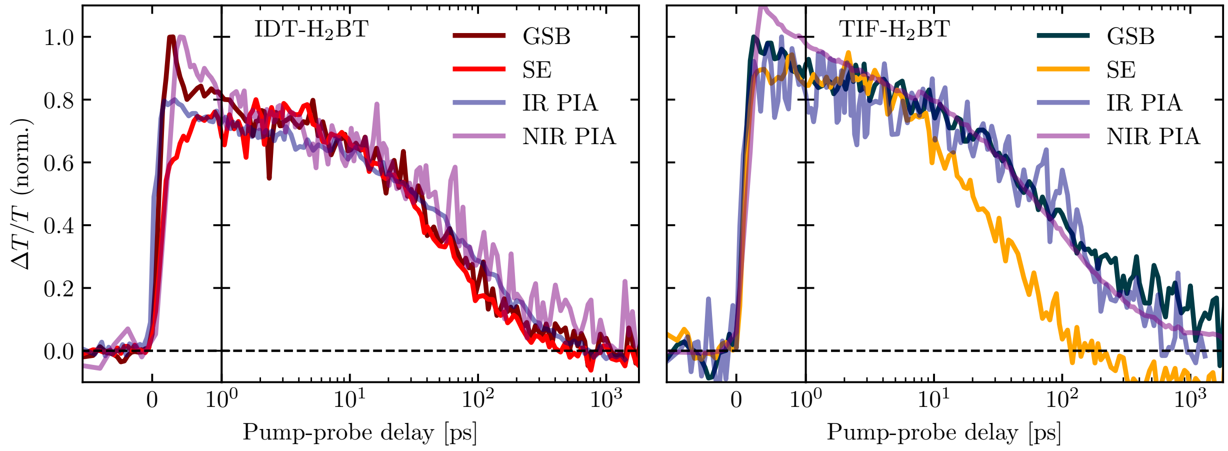

Supplementary Figure 8: Evolution kinetics of TA spectral features using $n_0 = 5 \times 10^{17}$cm^{-3}.

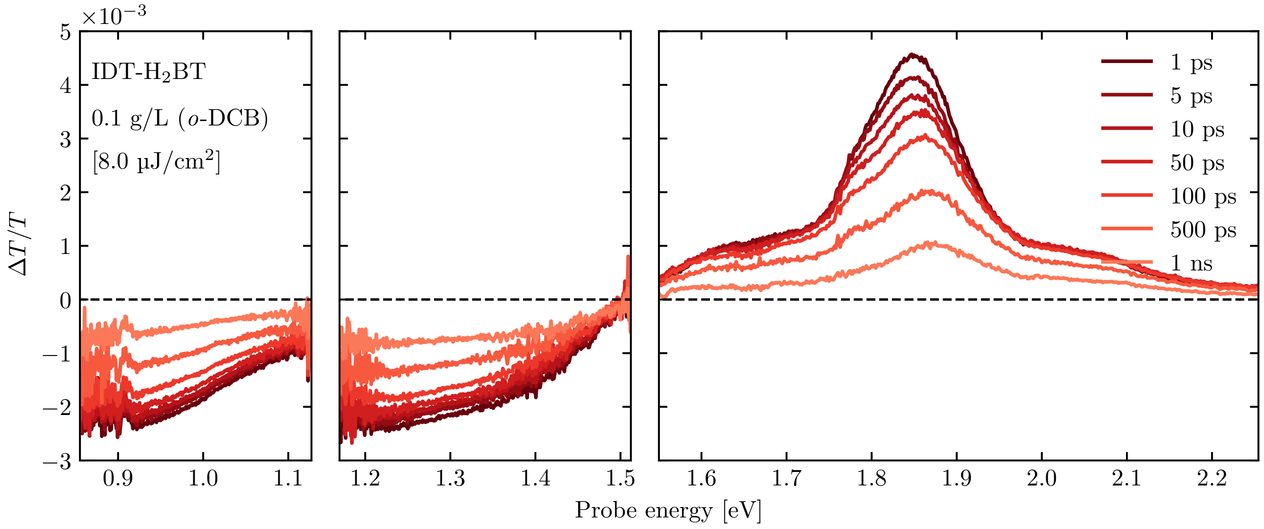

Supplementary Figure 9: TA spectral evolution from 1 ps–1 ns for IDT-H_2BT (0.1 g/L in *o*-DCB).

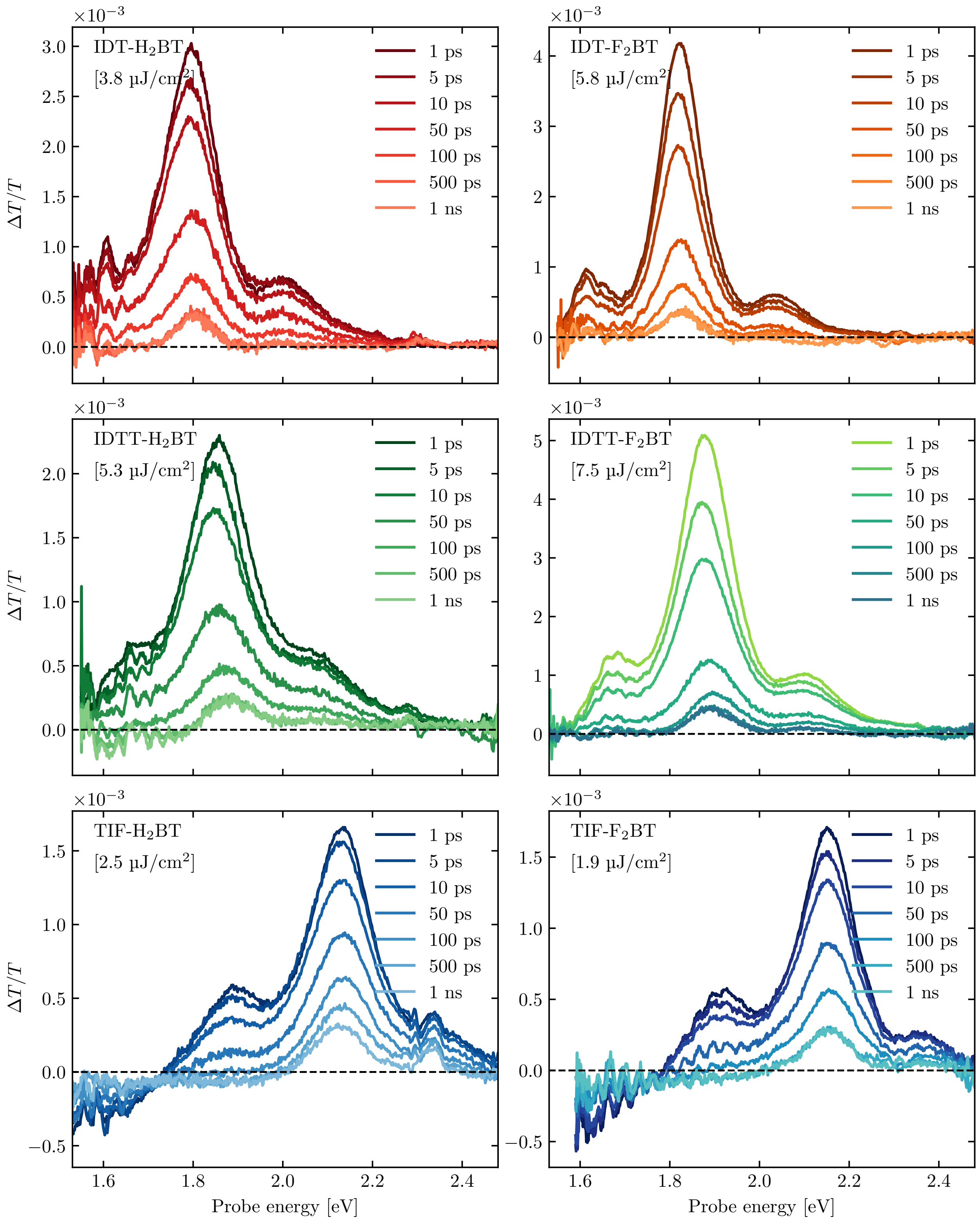

Supplementary Figure 10: TA spectral evolution from 1 ps−1 ns. For each sample, $n_0 = 5 \times 10^{17}$ cm^{-3}.

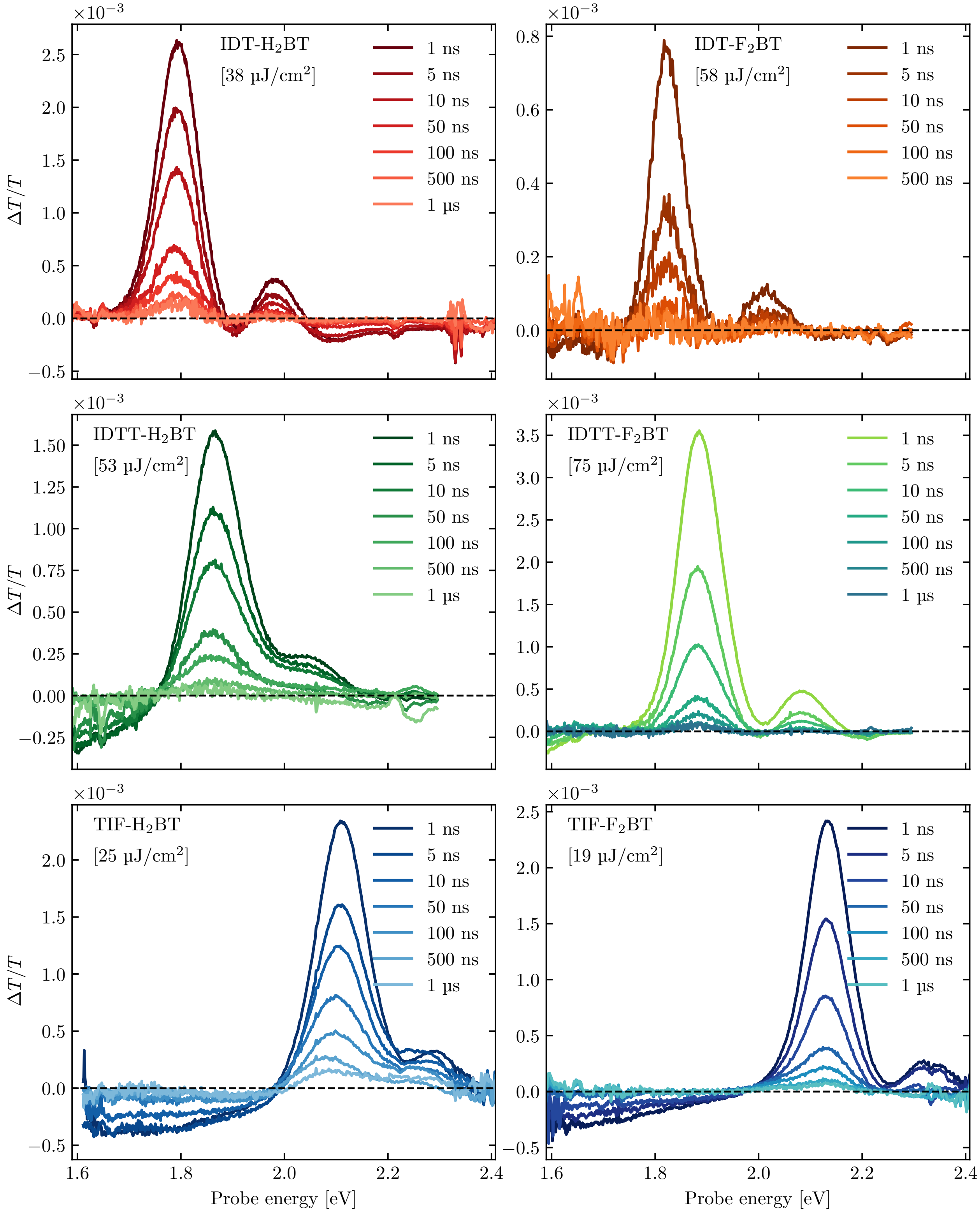

Supplementary Figure 11: TA spectral evolution from 1 ns−1 µs. For each sample, $n_0 = 5 \times 10^{18}$ cm^{-3}.

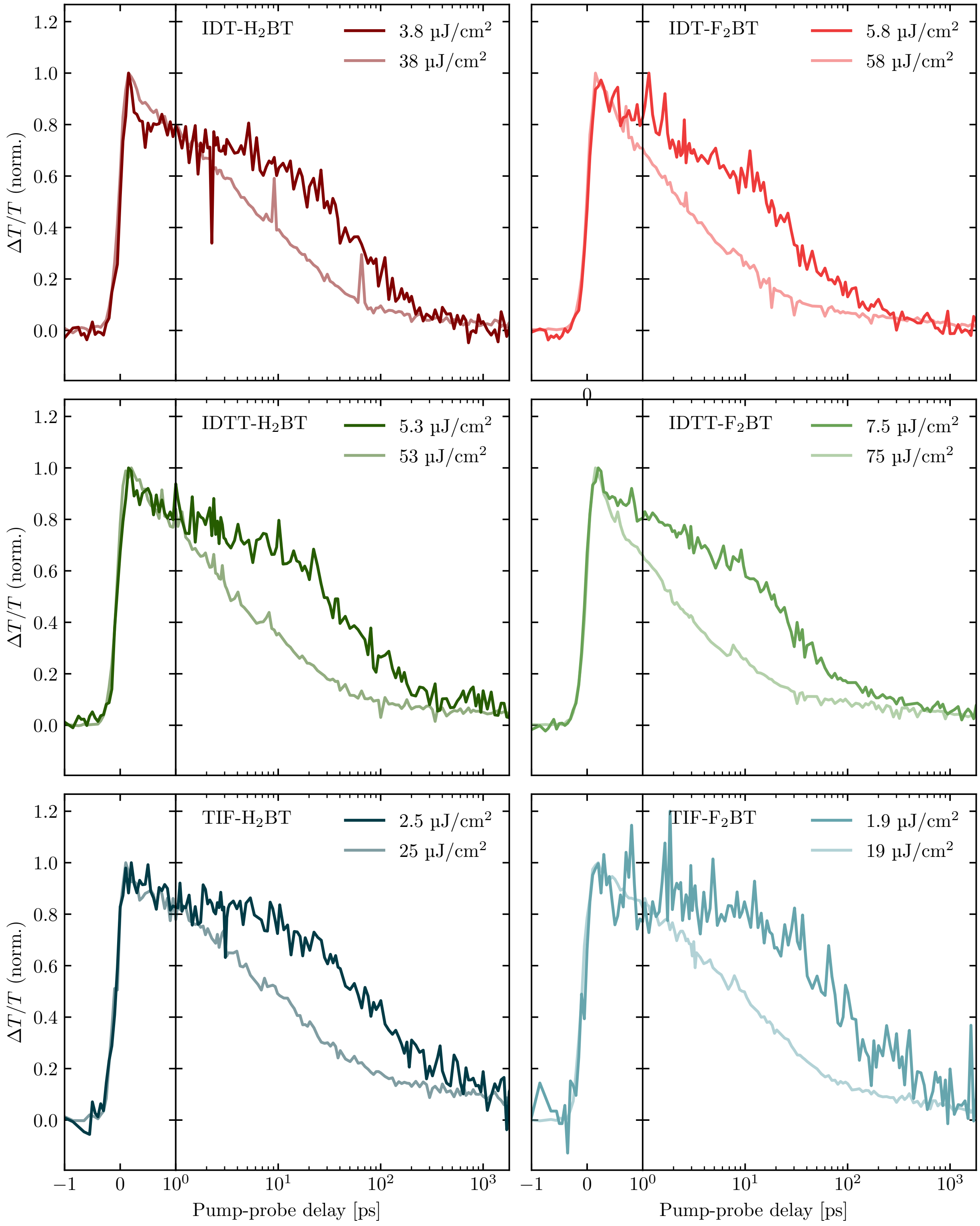

Supplementary Figure 12: Kinetic of the GSB (normalised to the maximum) for different fluence. For each polymer, $n_0 = 5 \times 10^{17}$ cm^{-3} (dark) and 5×10^{18} cm^{-3} (light).

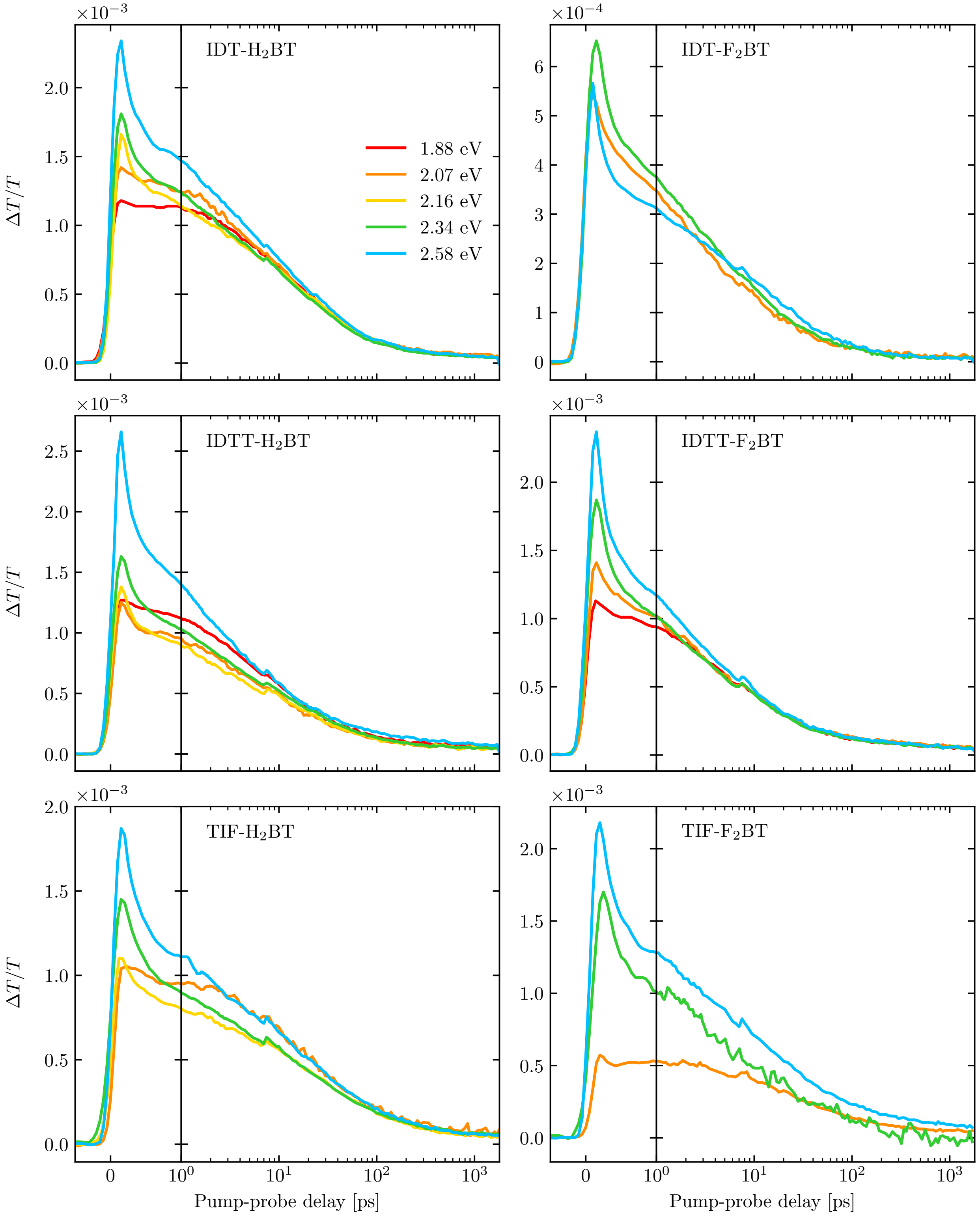

Supplementary Figure 13: Kinetic of the GSB for different pump wavelength. For each polymer, $n_0 \sim 10^{18}$ cm^{-3}.

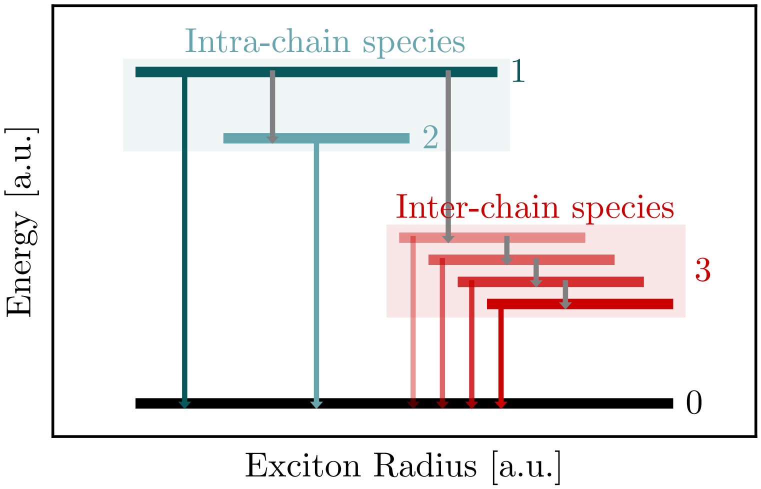

Supplementary Figure 14: Energy level diagram separating intra- and interchain excitonic species.

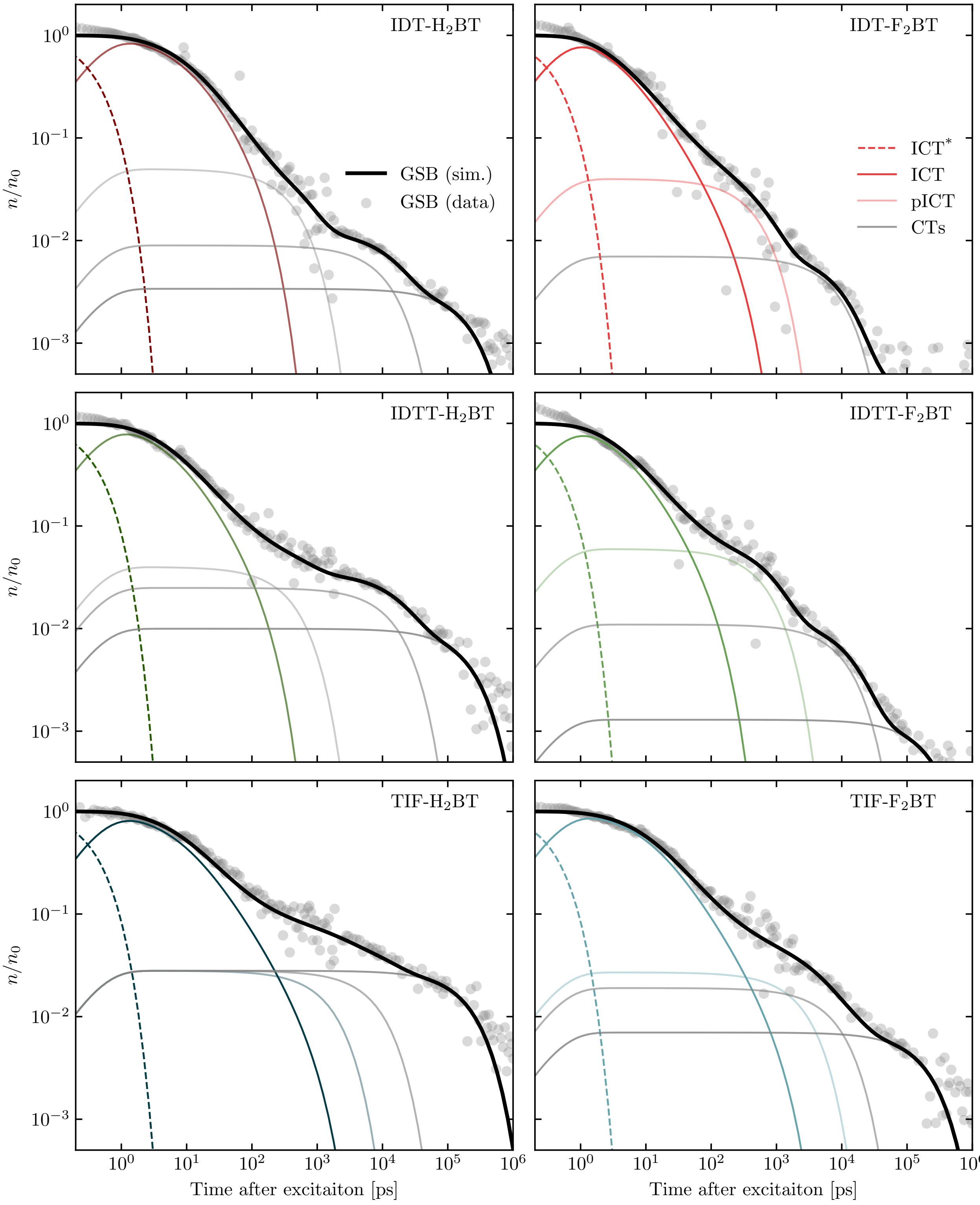

Supplementary Figure 15: Excited-state dynamics and GSB fit for different materials. Here, $E_{\text{pump}} = 2.33$ eV and $n_0 = 5 \times 10^{18}$ cm^{-3}.

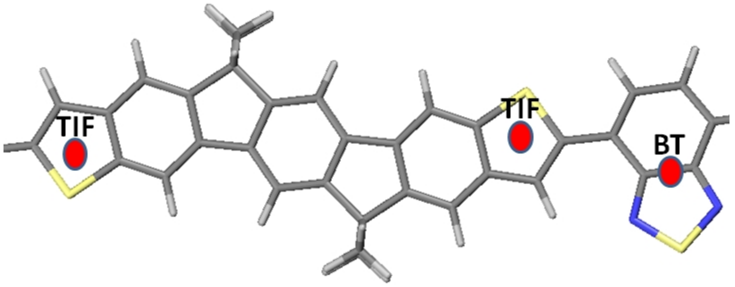

Supplementary Figure 16: Reference geometries for radial distribution function calculation.

Supplementary Tables

Supplementary Table 1: Energy gap, PLQE, and mobility values for polymers. Values from this study marked *

Polymer	E_g [eV]	Φ	μ [cm^2/Vs]	$\Phi \cdot \mu$ [cm^2/Vs]
DPP-DTT	1.4	0.002*	2*	4×10^{-3}
PCPDTBT	1.85	0.06*	3.7×10^{-3} [1]	2.2×10^{-4}
pBTTT	1.9	0.02*	1*	2×10^{-2}
P3HT	1.98	0.04 [2]	0.1 [3]	4×10^{-3}
CN-PPV	2.13	0.35*	2×10^{-5} [4]	7×10^{-6}
F8-TBT	2.2	0.41*	3×10^{-4} [5]	1.2×10^{-4}
MDMO-PPV	2.21	0.08 [6]	4×10^{-4} [7]	3.2×10^{-5}
MEH-PPV	2.25	0.12*	5×10^{-5} [8]	6×10^{-6}
F8-T2	2.29	-	5×10^{-3} [8]	-
F8-BT	2.4	0.5 [2]	0.01*	5×10^{-3}
Y80F8:20F5	2.9	0.7 [9]	0.037 [9]	2.6×10^{-2}
S70F8:20F5	2.9	0.6 [9]	0.027 [9]	1.6×10^{-2}
PFO	2.91	0.5 [9]	3×10^{-4} [9]	1.5×10^{-4}
IDT-H_2BT	1.70	0.017*	1.71*	2.9×10^{-2}
IDT-F_2BT	1.73	0.02*	1.25*	2.5×10^{-2}
IDTT-H_2BT	1.79	0.02*	0.63*	1.3×10^{-2}
IDTT-F_2BT	1.82	0.02*	0.69*	1.4×10^{-2}
TIF-H_2BT	1.99	0.15*	2.4*	3.6×10^{-1}
TIF-F_2BT	2.01	0.19*	0.29*	5.5×10^{-2}

Supplementary Table 2: Extracted effective mobility for polymers in this study.

Polymer	$\mu'_{\text{lin.}}$ [cm^2/Vs]	$\mu'_{\text{sat.}}$ [cm^2/Vs]	M_n [kDa]	M_w [kDa]	PDI
IDT-H_2BT	1.4	1.7	110	290	2.6
IDT-F_2BT	0.86	1.3	45	77	1.7
IDTT-H_2BT	0.58	0.62	67	151	2.3
IDTT-F_2BT	0.58	0.69	63	200	2.7
TIF-H_2BT	2.3	2.4	85	116	1.4
TIF-F_2BT	0.29	0.093	67	98	1.5

Supplementary Table 3: Fluences and excitation densities for matched $\Delta T/T$ signal.

Material	λ_{pump} [nm]	f_{pump} [J/cm^2]	n_0 [cm^{-3}]	$\bar{n}_0 \pm \sigma$ [cm^{-3}]
IDT-H_2BT	660	1.7×10^{-6}	1.7×10^{18}	2.6×10^{18} ±29 %
	600	3.7×10^{-6}	3.0×10^{18}	
	575	5.5×10^{-6}	3.7×10^{18}	
	530	4.1×10^{-6}	1.7×10^{18}	
	480	1.1×10^{-5}	2.7×10^{18}	
IDT-F_2BT	600	5.7×10^{-6}	5.8×10^{18}	3.7×10^{18} ±40 %
	530	6.2×10^{-6}	3.1×10^{18}	
	480	7.8×10^{-6}	2.2×10^{18}	
IDTT-H_2BT	660	4.4×10^{-6}	5.0×10^{18}	4.8×10^{18} ±22 %
	600	5.6×10^{-6}	5.1×10^{18}	
	575	8.1×10^{-6}	6.1×10^{18}	
	530	5.8×10^{-6}	2.8×10^{18}	
	480	1.7×10^{-5}	4.8×10^{18}	
IDTT-F_2BT	660	4.2×10^{-6}	3.3×10^{18}	4.0×10^{18} ±17 %
	600	7.3×10^{-6}	5.0×10^{18}	
	530	8.0×10^{-6}	4.5×10^{18}	
	480	1.7×10^{-5}	4.3×10^{18}	
TIF-H_2BT	600	5.5×10^{-6}	2.8×10^{18}	2.6×10^{18} ±23 %
	575	4.6×10^{-6}	3.2×10^{18}	
	530	2.7×10^{-6}	1.6×10^{18}	
	480	6.5×10^{-6}	2.6×10^{18}	
TIF-F_2BT	600	4.6×10^{-6}	1.6×10^{18}	1.7×10^{18} ±30 %
	530	2.1×10^{-6}	1.2×10^{18}	
	480	6.5×10^{-6}	2.4×10^{18}	

Supplementary Table 4: Measured parameters for the GSB of different polymers.

Polymer	k_1 [s^{-1}]	k_2 [s^{-1}]	k_3 [s^{-1}]
IDT-H_2BT	2.5×10^{12}	1.1×10^{10}	-
IDT-F_2BT	2.5×10^{12}	6.4×10^{9}	1.8×10^{9}
IDTT-H_2BT	2.5×10^{12}	1.0×10^{10}	-
IDTT-F_2BT	2.5×10^{12}	1.4×10^{10}	1.3×10^{9}
TIF-H_2BT	2.5×10^{12}	1.8×10^{9}	5.3×10^{8}
TIF-F_2BT	2.5×10^{12}	1.4×10^{9}	3.4×10^{8}

Supplementary Table 5: Fit parameters for the GSB of different polymers.

Polymer	$k_2^{(2)}$ [s^{-1}]	k_3 [s^{-1}]	$\phi_{1\to3}$	k_4 [s^{-1}]	$\phi_{1\to4}$	k_5 [s^{-1}]	$\phi_{1\to5}$	ϕ_{IS}
IDT-H_2BT	1.0×10^{11}	2.0×10^{9}	0.05	7.1×10^{7}	0.009	4.2×10^{6}	0.0034	0.0624
IDT-F_2BT	2.9×10^{11}	-	0.04	1.4×10^{8}	0.007	4.0×10^{6}	0.0005	0.0475
IDTT-H_2BT	1.8×10^{11}	2.0×10^{9}	0.05	5.5×10^{7}	0.025	4.0×10^{6}	0.01	0.075
IDTT-F_2BT	2.5×10^{11}	-	0.06	7.6×10^{7}	0.011	4.0×10^{6}	0.0013	0.0723
TIF-H_2BT	1.3×10^{11}	-	0.028	1.0×10^{8}	0.028	4.0×10^{6}	0.028	0.084
TIF-F_2BT	9.1×10^{10}	-	0.027	1.0×10^{8}	0.019	4.3×10^{6}	0.007	0.053

Supplementary Notes

Supplementary Note 1: Mobility extraction in polymer semiconductors

Interpreting OFET data with the behaviour expected in an ideal MOSFET is a powerful tool to probe the charge transport behaviour in semiconducting materials. Comparison with analytical equations gives information about the mobility, trapping mechanism, and disorder in the transport DOS by measuring the transconductance as a function varying charge carrier accumulation and temperature. At a constant temperature, the mobility can theoretically be extracted in the linear (lin.) regime, where $|V_{\mathrm{DS}}| < |V_{\mathrm{GS}} - V_{\mathrm{Th}}|$,

$$I_{\mathrm{DS}} = \mu_{\mathrm{lin.}} C_{\mathrm{ox.}} \frac{W}{L} \left((V_{\mathrm{GS}} - V_{\mathrm{Th}}) V_{\mathrm{DS}} - \frac{(V_{\mathrm{DS}})^2}{2} \right) \tag{1}$$

where there is a linear dependence of I_{DS} on V_{GS}. On the other hand, the saturation (sat.) regime has $|V_{\mathrm{DS}}| > |V_{\mathrm{GS}} - V_{\mathrm{Th}}|$, and

$$I_{\mathrm{DS}} = \mu_{\mathrm{sat.}} C_{\mathrm{ox.}} \frac{W}{2L} (V_{\mathrm{GS}} - V_{\mathrm{Th}})^2 \tag{2}$$

and I_{DS} no longer depends on $|V_{\mathrm{DS}}|$. Within this formulation, $\mu_{\mathrm{lin.}}$ and $\mu_{\mathrm{sat.}}$ describe the charge carrier mobility of the system and should agree, in principle.

However, applying these equations in spite of a deviation from ideal MOSFET behaviour can lead to extraction of erroneous mobility values. The origin of common problems in OFETs arise from contact resistance, high threshold voltages and hysteresis due to charge-carrier trapping. These non-idealities can all lead to the overestimation of the mobility. While hysteresis and high-threshold voltages are typically easy to diagnose in devices, contact resistance (which can be gate-voltage dependent[10]) can lead to current crowding effects[11], which is often less obvious.

We observe that using gold electrodes (with a work function of ~ -5.4 eV), a bottom-contacted architecture, and appropriate solution processing conditions that incorporate a small concentration of a small molecular additive into the film (described below) can remove these effects in most of our materials. In particular, using residual solvents with a high boiling point, such as 1,2-dichlorobenzene, that remain in the film after spin-coating and annealing, or a small quantity of non-doping molecular additive, such as tetracyanoquinodimethane (TCNQ) as described by Nikolka, Nasrallah, *et al.*,[12] leads to improved device performance, stability, and contact resistance. These were not found to

affect or degrade the photophysical properties. While in the MOSFET formulation the mobility is a constant and $\mu_{\text{lin.}} = \mu_{\text{sat.}}$, rearrangement of the MOSFET equations allows for the evaluation of a mobility which, in general, has a dependency on V_{GS};

$$\mu_{\text{lin.}}(V_{\text{GS}}) = \frac{1}{V_{\text{DS}}} \frac{1}{C_{\text{ox.}}} \frac{L}{W} \left(\frac{\partial I_{\text{DS}}}{\partial V_{\text{GS}}} \right) \tag{3}$$

in the linear regime, and

$$\mu_{\text{sat.}}(V_{\text{GS}}) = \frac{1}{C_{\text{ox.}}} \frac{2L}{W} \left(\frac{\partial \sqrt{I_{\text{DS}}}}{\partial V_{\text{GS}}} \right)^2 \tag{4}$$

in saturation. By calculating the mobility in this way, we are able to clearly diagnose ideal a non-ideal MOSFET-like behaviour in the characteristics. Together with an extraction of V_{Th} using extrapolation of the linear region of the $\sqrt{I_{\text{DS}}} - V_{\text{GS}}$ graph in the limit that $|V_{\text{GS}}| \gg |V_{\text{Th}}|$ to $I_{\text{DS}} = 0$ A (where the device is operated in saturation), we have a full picture of whether the device is well-described by the MOSFET model.

Our fabrication technique, incorporating incorporating molecular additives which passivate water induced traps, is able to improve charge injection significantly. The uniformity and contact resistance of a range of polymers can be optimised in this way.[12] This said, the very deep lying HOMO levels ($5.7-5.8$ eV) of TIF-H_2BT, TIF-F_1H_1BT and TIF-F_2BT inevitably result in a larger injection barrier as compared to IDT-H_2BT (with a HOMO of 5.3 eV). Furthermore, their deeper HOMO levels make the TIF polymers more sensitive to trapping of polarons on the polymer backbone by residual water molecules. This makes it even more important to incorporate molecular additives into the films than in IDT-H_2BT. We attempted to extract the contact resistance using the transfer length method for TIF-F_1H_1BT and although we extract a contact resistance value that is only slightly higher than in IDT-H_2BT (fabricated using our additive route as well), the device uniformity is not good enough to extract precise values (**Supplementary Figure 1**). The same applies to the polymers TIF-H_2BT and TIF-F_2BT.

In **Supplementary Figure 2**, we show the OFET data used to determine the p-type mobility of polymers in this study. In all devices, we use channel architectures with $L = 20$ µm and $W = 1$ mm dimensions. A polymer film of $20 - 40$ nm was cast, followed by a Cytop gate dielectric of thickness ~ 500 nm. This leads to a gate capacitance of 37 µF/m^2. For each material (in a different colour), we show the transfer characteristics (top) in the linear (dark) and saturation (light) regimes. Below this, we show the extraction of $\mu_{\text{lin.}}$ and $\mu_{\text{sat.}}$ as a function of V_{GS} (using **Supplementary Equations 3**

and **4** respectively). IDT-H_2BT is most well-behaved with respect to the device problems detailed above, however every material in this study exhibits a degree of non-ideal behaviour. In this family of materials, this is exemplified by a V_{GS}-dependent μ, and a disagreement between $\mu_{\mathrm{lin.}}$ and $\mu_{\mathrm{sat.}}$. We find this behaviour to be very common in disordered organic semiconductor systems, and that these effects can be reduced substantially by optimising the device preparation conditions. Nonetheless, it is important to have a robust and uniform methodology for mobility extraction, and therefore we define the effective mobility (μ') by rearranging **Supplementary Equations 1** and **2** to reflect the average current output and architecture of the device. It can be shown that

$$\mu'_{\mathrm{lin.}} = \frac{1}{V_{\mathrm{DS}}}\frac{1}{C_{\mathrm{ox.}}}\frac{L}{W}\left(\frac{I_{\mathrm{DS}}^{\mathrm{max.}} - I_{\mathrm{DS}}^{0}}{V_{\mathrm{GS}}^{\mathrm{max.}}}\right) \tag{5}$$

in the linear regime, and

$$\mu'_{\mathrm{sat.}} = \frac{1}{C_{\mathrm{ox.}}}\frac{2L}{W}\left(\frac{\sqrt{I_{\mathrm{DS}}^{\mathrm{max.}}} - \sqrt{I_{\mathrm{DS}}^{0}}}{V_{\mathrm{GS}}^{\mathrm{max.}}}\right)^2 \tag{6}$$

in saturation, where $I_{\mathrm{DS}}^{\mathrm{max.}}$ is the experimental maximum on-current (observed at $V_{\mathrm{GS}} = V_{\mathrm{GS}}^{\mathrm{max.}}$), and I_{DS}^{0} is the off-current (observed at $V_{\mathrm{GS}} = 0$ V). This methodology provides a conservative lower bound on the true mobility, and is helpful when the application of **Supplementary Equations 3** and **4** is inappropriate. We find this to be the case in almost all circumstances, and this methodology will be discussed in more detail in a forthcoming paper co-authored by one of us. The effective mobility is summarised in **Supplementary Table 2**. When quoting the mobility of a material in this work, we use the maximum of the two values presented in this table.

Another important factor in diagnosing poor device performance can be achieved by using the output transconductance characteristics. It is here that contact resistance often becomes visible, and can lead to a suppression of I_{DS} near $V_{\mathrm{DS}} = 0$, and therefore a sigmoid shape in I_{DS} overall. We show the output characteristics for each material in this study in **Supplementary Figure 2** below the transfer characteristics. Appropriate choice of electrode materials and solution processing mean that we do not observe this in our devices, except for TIF-F_2BT, which has an exceedingly deep HOMO energy.

The different polymers exhibit some variations in molecular weight (**Supplementary Table 2**). For a related polymer (IDT-H_2BT with hexadecylbenzyl sidechains), we have investigated the molecular weight dependence in detail and found that a higher degree of polymerisation does not appreciably increase or decrease the mobility,[13] once the molecular weight exceeds about 20 kDa.

In the present work all polymers have reasonably high molecular weights above 45 kDa and we do not expect a significant dependence of the electrical and photophysical properties on molecular weight in this class of polymers.

As a final point, we note that after the device 'pinches off' (i.e. for $V_{DS} > V_{GS}$), I_{DS} should become constant ('saturate'). With our top-gate bottom-contact device architecture, and using a thin polymer film, we do not observe this behaviour. This is due to a back-channel effect formed at high $|V_{GS}|$ where accumulation near the un-grounded glass substrate leads to a second effective channel, and so I_{DS} continues to increase the applied $|V_{GS}|$. We have observed separately for IDT-H_2BT that fabricating a device on a doped-Si substrate, and grounding the substrate during the measurement decreases this effect substantially.[12]

Regarding μ extraction for TIF-H_2BT, despite the higher ionisation potential than IDT-H_2BT, the contact resistance remains sufficiently low and the output characteristics in **Figure 2d** lack a sigmoidal contact artefact near $V_{DS} = 0$ V. The increasing mobility of TIF-H_2BT, or superlinear $\sqrt{I_{DS}} - V_{GS}$ behaviour at high accumulations, is curious. While this has been attributed to a broad transport density of states[14, 15], or a Coulomb effect in semicrystalline polymers[16], its origin in these low-energetic disorder, near-amorphous polymers is unclear. We note that conventional extraction using a linear fit to $\sqrt{I_{DS}} - V_{GS}$ is inappropriate here and yields $\mu > 6$ cm^2/Vs, which is inconsistent with the increase of only ~ 50 % in on-current values between IDT-H_2BT and TIF-H_2BT in saturation. Instead, an effective mobility value of $\mu = 2.4$ cm^2/Vs is a conservative estimate, and the experimental current output is consistent with what current would be expected from an ideal MOSFET of this mobility.

Supplementary Note 2: Push-pull character and optical transitions

Push-pull character and the energy gap The degree of push-pull character and energetic disorder is of crucial importance in this work. Regarding the former, increasing the length of the donor unit but maintaining the same acceptor (i.e., either H_2BT or F_2BT) allows us to modulate the degree of push-pull character (and hence, E_g), as described in the main text. Substitution of H_2BT for F_2BT has been shown to increase the ionisation potential of the polymer (because of its increased electron-withdrawing properties) without substantially changing the lowest unoccupied molecular or-

bital (LUMO) energy in some similar systems.[17, 18]

The photoluminescence measurements involved optically pumping well above the E_g-edge ($E_{\text{pump}} = 3.05$ eV), and collecting time-integrated spectra using very low fluences ($f_{\text{pump}} \sim 2$ µJ/cm^2). These were normalised to their maximum in **Figure 2f**.

H-aggregation in TIF-H_2BT The optical transitions in conjugated polymers often couple strongly to C=C ring stretching modes ($\sim 1200-1400$ cm^{-1}, i.e. $\sim 0.15-0.20$ eV), and we observe a vibronic progression of decreasing PL intensity arising from $S_1(\nu = 0) \rightarrow S_0(\nu = m)$ transitions (hereunder $I_{0\rightarrow m}$) with $m = 0, 1, 2$ at 1.73, 1.59, and 1.44 eV in IDT-H_2BT and 1.99, 1.85, and 1.70 eV in TIF-H_2BT, respectively. In the absence of interchain interactions, the Franck-Condon model applies,[19] and,

$$I_{0\rightarrow m} \sim (nE_{\text{photon}})^3 \cdot \frac{S^m \exp(-S)}{m!} \tag{7}$$

where n is the refractive index, and S is the Huang-Rhys factor. However, $I_{0\rightarrow 0}/I_{0\rightarrow 1}$ can be suppressed by both exciton localisation[20] and H-aggregation[21]. $I_{0\rightarrow 0}/I_{0\rightarrow 1}$ is considerably suppressed in TIF-H_2BT compared to IDT-H_2BT. Separately, we estimate the exciton diffusion length to be 12.8 ± 1.9 nm in TIF-H_2BT using a method described elsewhere;[22] an improvement over 7.1 ± 1.1 nm for IDT-H_2BT. Therefore, TIF-H_2BT has both increased exciton delocalisation and more pronounced H-aggregate character compared to IDT-H_2BT. This suggests that the more extended conjugation and relatively smaller sidechain density of this polymer facilitate more pronounced interchain interactions at close-crossing points of the polymer chains, which are fully consistent with the observed increase in field-effect mobility. A similar comparison has been made in poly(2-methoxy-5-(2-ethylhexyloxy)-1,4-phenylenevinylene) (MEH-PPV)[23], and poly(3-hexylthiophene-2,5-diyl) (P3HT)[24].

Supplementary Note 3: Spectroscopic characterisation of interchain species

Interchain species in IDTT-F_2BT In order to verify the interchain nature of the additional luminescence pathway in these materials, we performed additional experiments on IDTT-F_2BT, for whom the second (redshifted) PL pathway is spectrally resolved from the ICT. These experiments are summarised in **Supplementary Figure 3**, and used $E_{\text{pump}} = 3.05$ eV and low fluence < 2 µJ/cm^2.

Firstly, we note in **Supplementary Figure 3** (top left) that the second PL pathway is present in IDTT-F_2BT, but not in IDTT-H_2BT. More accurately, this implies that a second PL pathway may be weaker, or not spectrally resolved for IDTT-H_2BT. However, tr-PL measurements separately confirm that IDTT-H_2BT has no additional long-lived PL decay kinetic (with $\tau_{\mathrm{PL}} > 250$ ps) like for IDTT-F_2BT. Secondly, we note that this emissive pathway is not present for IDTT-F_2BT in dilute solution (top right). While the PL lineshape of IDTT-F_2BT in 0.1 g/L 1,2-dichlorobenzene (*o*-DCB) is broader, we do not observe an increase in signal near 1.5 eV. This suggests that the peak below 1.5 eV arises in some way from the aggregation.

To confirm this picture, we added a aliquots of poor solvent for IDTT-F_2BT to a solution (1 g/L, *o*-DCB) to promote aggregation (bottom left). Compared to the neat IDTT-F_2BT solution spectrum (dark purple), we do not see a substantial change in the PL spectrum below 1.5 eV until the analyte solution is comprised of equal parts *o*-DCB and IPA (1 : 1) by volume (light purple). With additional IPA, we observe another peak appearing in the PL below 1.5 eV, and this peak becomes stronger towards the point where there is a three-fold excess of IPA (1 : 3) in the analyte solution (light green). This experiment confirms that the peak below 1.5 eV arises from the aggregation, since its intensity can be controlled by increasing the aggregation in solution.

Finally, in order to show that this pathway specifically involves multiple polymer chains, we blend IDTT-F_2BT with PMMA (bottom right). Between the neat IDTT-F_2BT film (black) and a film with a 10-fold excess of PMMA (dark purple) by weight, we see a small suppression of the emission pathway below 1.5 eV. However, this peak is still strong, and ~ 50 % of the ICT maximum. Upon increasing the PMMA weight excess to 100×, this peak is suppressed further to $< 30\%$ of the ICT maximum. Thereafter, the decrease in this peak is rapid, and by an excess of 200×, this peak is < 10 % of the ICT maximum. This experiment confirms the 'multiple polymer chain' origin of this peak, and suggests that when polymer chains cannot come close together, this emissive pathway is shut down. Owing to its redshifted energy compared to the on-chain ICT, and its substantially increased photoluminescence decay lifetime (discussed in the main text), this recombination pathway is likely to involve an interchain charge transfer.

By using an OLED architecture, it is possible to measure the electroluminescence (EL) of the polymer to improve our understanding of this sub-E_g state. The EL is compared to the PL in **Sup-**

plementary Figure 4. At low current densities, most of the EL comes from the interchain species state. This is consistent with Kasha's rule, and recombination is most likely from the lowest state energy in the JDOS. This mechanism is likely to involve exciton diffusion to these sites. At higher current densities by $\sim 12\times$, we observe that Kasha's rule is seemingly violated, and the ICT peak at 1.8 eV increases in relative intensity to the interchain species. This behaviour suggests increased relative exciton occupancy of the ICT state as all available interchain species become occupied. In the steady state, this is facilitated by a substantially longer interchain species recombination lifetime. At the same time, the blue edge of the ICT transition increases as all lower energy ICT microstates become filled, and additional excitons fill higher energy microstates to avoid double-occupancy. There is good spectral agreement between the EL and PL.

Linewidths of IS absorption and luminescence Comparing PDS (absorption) and luminescence spectra in **Figure 2e** and **f** of the main text, respectively, we discuss that distinct absorption features are clearly visible for IDTT-F_2BT in the sub-E_g regime. These are also observed in TIF-H_2BT and TIF-F_2BT. In the $1.3 - 1.9$ eV range, these absorptions have a similar absorption cross-section for IDTT-F_2BT and TIF-H_2BT, with latter absorption centred around 1.75 eV. In addition, for TIF-H_2BT, we resolve a second sub-E_g absorption feature, centred at 1.35 eV, with a $\sim 5\times$ weaker absorption strength. The interpretation of the second sub-E_g absorption features is complicated, however, by a potential contribution from accidental doping of the polymer in this energy range. We note that the hole polaron has a charge-induced absorption in the $1.3 - 1.6$ eV region (**Supplementary Figure 5**), and that in purified batches of TIF-H_2BT primarily the second sub-E_g feature can be reduced in intensity.

To investigate the origin of the strong absorption feature in the sub-E_g regime for TIF-H_2BT, we compare the absorption of a purified sample and an unpurified sample in **Supplementary Figure 5** (top). Both samples are nearly indistinguishable above the E_g edge (which is the same). However, in the sub-E_g regime, the unpurified sample (blue) exhibits a broad absorption feature between $1.3-1.9$ eV. By contrast, the purified sample (grey) exhibits two distinct and spectrally resolved absorption features, centred around 1.40 eV and 1.75 eV. We posit that the difference between the batches, and hence absorption spectra, is a degree of p-type doping leading to an additional broad absorption spectrum of the hole polaron in this range.

To confirm this assignment, we independently measure the spectrum of the hole polaron using charge accumulation spectroscopy (CAS). Here, we use a transistor architecture (with the same channel length), and measure the differential transmission with/without gate bias (V_{GS}). Since V_{GS} is proportional to the number of accumulated of charges, the induced absorption spectrum of hole polarons is inferred as a negative feature in the $\Delta T/T$ plot in **Supplementary Figure 5** (bottom). The precise details of the experiment are reported elsewhere.[16] Below 1.6 eV, we observe a strong signal due to the hole polaron, and its absorption spectrum is broad in the range of $1.3 - 1.6$eV. This suggests that unintentional doping could potentially make a contribution to the PDS signal in this energy range and could obscure the presence of absorbing interchain species in the same band. Electroabsorption is responsible the derivative-like feature at the E_g-edge and near 3.1 eV.

A subtle fluence-dependency of the luminescence spectra of IDT-H_2BT provides some evidence for the formation of luminescent interchain species as well. This is hard to quantify precisely with this methodology, since precise measurement of the recombination lifetime from the PL requires specialised ultrafast techniques and typically high fluence. In **Supplementary Figure 6**, we measure the fluence dependence of the PL spectra. We observe in these polymers that PL can be quenched efficiently by non-radiative bimolecular exciton recombination, and in IDT-H_2BT, $\Phi \sim 4.5 \times 10^{-3}$ at $f_{\mathrm{pump}} \sim 70$ µJ/cm^2. However, we observe for all of our polymers that the formation quantum efficiency of interchain species does not depend on the fluence. This is discussed in detail later. Therefore, by increasing the fluence, it should be possible to decrease the PLQE ϕ_{PL} of the ICT state, and observe an increase in relative PL from any emissive interchain species.

The PL measurements in **Supplementary Figure 6** show this experiment for IDT-H_2BT, using $E_{\mathrm{pump}} = 3.10$ eV and fluences as labelled, integrated on-chip in time, and normalised to the maximum. For a dilute solution of IDT-H_2BT (left), we do not resolve a change in the PL spectra with increased fluence. For a thin film (right), we resolve a slight difference around 1.6 eV in the normalised spectra. By subtracting the low-f spectrum from the high, we observe clearly an additional contribution to the PL which is otherwise hidden under the strong ICT PL. Furthermore, this peak is redshifted with respect the the ICT, but extremely broad. By fitting a Gaussian lineshape, we recover that this peak is centred near 1.6 eV. and has a full-width at half-maximum (FWHM) of ~ 0.65 eV. Therefore, this could potentially be due to interchain species in IDT-H_2BT. However, in

this case, the emission is much weaker in intensity than the ICT, and more similar in recombination rate, making a more detailed analysis challenging.

Spectral decomposition using TCSPC The methodology for spectral decomposition using analysis of the PL lifetimes is robust even when the two luminescent species have overlapping spectra, and this procedure was repeated for IDT-F_2BT (τ_{IS} = 560 ps, τ_{ICT} < 250 ps), TIF-H_2BT (τ_{IS} = 1.9 ns, τ_{ICT} = 560 ps) and TIF-F_2BT (τ_{IS} = 3.0 ns, τ_{ICT} = 700 ps). From the genetic algorithm we extract PL spectra of both the prompt ICT PL and the red-shifted longer-lived interchain emission, which are shown in normalised form in **Figure 3a**. A limitation of this methodology is that we are unable to distinguish ICT and IS luminescence when τ_{ICT} and τ_{IS} are both less than 250 ps. As a result we were unable to directly observe luminescent interchain species in IDT-H_2BT and IDTT-H_2BT, which have weak PL and a very short lifetime τ_{ICT} < 250 ps.[25]

The PL lifetimes increase with E_g, as predicted by the energy gap law. However, there is also strong variation in the spectral linewidth of the IS luminescence between the materials. TIF-H_2BT has the broadest IS transitions with a FWHM of > 0.25 eV, compared to < 0.2 eV in IDTT-F_2BT. The FWHM of a PL transition is related to the width of JDOS weighted by the oscillator strength, and the broadband luminescence of TIF-H_2BT and TIF-F_2BT suggests a broader energetic distribution of luminescent IS microstates compared to IDTT-F_2BT. By comparison with the sub-E_g absorption, we also note that only aggregate microstates leading to high energy IS transitions are luminescent, and those below 1.5 eV rather lead to non-radiative recombination. This is represented diagrammatically in **Figure 3b**.

Ultrafast transiet grating PL of TIF-H_2BT Using ultrafast transient grating PL (E_{pump} = 2.34 eV, f_{pump} = 25 µJ/cm^2, giving an excitation density $n_0 = 5 \times 10^{18}$ cm^{-3}), we measure the PL decay of TIF-H_2BT between 200 fs (the temporal resolution of the experiment) and 100 ps in **Figure 3c**. In the first 1 ps after excitation, the broad PL spectrum peaks at 2.08 eV. Over the next 5 ps, the spectrum narrows, decaying and redshifting to 2.04 eV, but the band below 1.85 eV does not decrease in intensity. Over the following 95 ps, the spectral maximum decays by a further 50 %, but the low-energy edge of the emission decays substantially less. At the earliest times, the ICT population we

observe has not reached energetic equilibrium - and is 'hot' (ICT*) - overlapping in energy with the maximum of the absorption band yielding a small Stokes shift. However, by 5 ps, the thermalisation of excitons is complete and the ICT* population has transferred to the luminescent ('equilibrated') ICT population. Separately, we have observed similar dynamics for IDT-H_2BT.[13] Interestingly, at the earliest times, the luminescence band below 1.85 eV, which contains the IS luminescence maximum, is already formed and does not redshift during ICT* population transfer. By integrating the shaded spectral windows, we show the normalised decay kinetics of three colour-corresponding energy bands in **Figure 3e**. These bands are chosen to minimise signal mixing, and correspond to the ICT* (dark grey), ICT (cyan), and IS (sky blue) luminescence. To within 100 fs, all three bands rise at the same time. The decay of the ICT* with a lifetime of 400 fs tracks its thermalisation to the ICT and IS states. After 1 ps, this converges to the ICT decay kinetics, which decreases over the following 100 ps. The interchain species decays with a longer lifetime than ICT. Importantly, this state has emission at very early times, and since the IS decay kinetics deviates within 400 fs from the ICT, we conclude that the interchain species must be formed at least as fast as the ICT* relaxation.

Supplementary Note 4: Transient absorption spectroscopy

Spectral features and time evolution In order to discuss how the TA spectra give us information about the population of excited state species, we show the TA spectral evolution for IDT-H_2BT and TIF-H_2BT thin films over the first 1 ns after the pump pulse in **Supplementary Figure 7**. Here, pump photons had $E_{\text{pump}} = 2.33$ eV and the fluence chosen gave an initial excitation density of 5×10^{17} cm^{-3}. We show the transient differential transmission spectrum over three broadband regions: IR (left), NIR (middle), and visible (right).

There is a high degree of similarity between the TA spectra for these materials. For IDT-H_2BT (TIF-H_2BT), we see the GSB above 1.70 eV (2.05 eV) which overlaps spectrally with the steady-state absorption spectrum in the main text. Accordingly, this has a vibronic progression at higher energies, and the 0-1 peak is at 2.00 eV (2.35 eV). Similarly, we see SE at 1.60 eV (1.9 eV), and by comparison with the steady-state PL spectrum, this peak is most likely the 0-1 vibronic peak of the SE, and therefore, the largest peak at 1.80 eV (2.15 eV) is likely to contain signal from the 0-0 of both GSB and SE. As the SE decays to zero, and the 0-1 GSB peak at 2.00 eV (2.35 eV) increases in intensity relative to the 0-0 GSB peak at 1.80 eV (2.15 eV). Finally, below 1.4 eV (1.7 eV), we see a negative

signal due to the absorption of the photoexcited states (PIA). In fact, this PIA is very broad, and extends well into the visible band, decreasing the apparent $\Delta T/T$ of the GSB to below zero towards 1 ns for IDT-H_2BT.

In the PIA, we resolve two broad absorption signatures which have their own lifetimes. This is a clear indication that they belong to the absorption of different excited state species. In IDT-H_2BT, the PIA seems to have two humps - one in the NIR ($1.2-1.4$ eV, with SE signal of the 0-2 vibronic peak > 1.4 eV), and one in the IR, with the $\Delta T/T$ increasing below 1.05 eV. These humps go to zero on subtly different timescales. The IR PIA goes to zero by 1 ns, and the peak intensity at each timestep correlates strongly with the SE. It is likely that while we do not fully resolve this peak, we observe the blue edge of the PIA of excitons in the ICT state. By contrast, the NIR hump has very similar dynamics - also correlating strongly in intensity with the SE until ~ 1 ns. However, in this case, when the SE has finished, there is still some PIA signal left which does not go to zero. In all likelihood, the PIA due to ICT excitons is below 0.8 eV, but whose tail extends into both IR and NIR bands, giving the similarity in decay to the SE. The remaining PIA signal above 1.2 eV extending into the visible band belongs to another species, and is likely to be an interchain species, judging by its longevity. For TIF-H_2BT, the picture is similar, except the PIA due to ICT excitons is considerably blueshifted to $0.9-1.6$ eV, and overlaps with the PIA due to interchain species above 1.2 eV. However, also in this case there is clear evidence for two species as the PIA signal around 1.2-1.3 eV decays almost completely to zero after 1 ns, while the signal around 1.4-1.5 eV retains a long lived components, which we attribute to the interchain species.

The decay kinetics, summarised in **Supplementary Figure 8**, tell the same story. For IDT-H_2BT (left), there is strong agreement between all the (normalised) kinetics of the spectral features. Importantly, while the IR PIA and SE (both arising from ICT excitons) decay to zero by ~ 1 ns, the NIR PIA and the GSB do not, since they arise from the small population of longer-lived interchain species. Additionally, it is important to note that all features rise within the instrument response of the setup (of ~ 100 fs), and therefore, both the ICT and interchain species are populated on ultrafast timescales. The picture for TIF-H_2BT (right) is similar. Here, owing to the overlap of NIR PIA and SE, the SE signal (yellow) appears to become negative by 200 ps, but simply decays to zero as before with the IR PIA since it arises from emissive ICT excitons. On the other hand, the interchain species PIA is weaker but longer lived (as before) and leads to good agreement between the GSB towards 1 ns.

To complete this picture, we show the TA spectra of IDT-H_2BT in dilute solution in **Supplementary Figure 9**. In this case, the exciton dynamics are comparable, except we are unable to resolve a population of interchain species by 1 ns. This is owing to the longer recombination lifetime of the ICT meaning there is still strong SE and strong IR PIA at this time.

To demonstrate the similarity of the exciton dynamics in these materials, we show the TA spectra for all of the materials studied here in **Supplementary Figure 10** at a low excitation density of 5×10^{17} cm^{-3} between 1 ps− 1 ns, and in **Supplementary Figure 11** at a higher excitation density of 5×10^{18} cm^{-3} between 1 ns− 1 µs.

Summary of exciton dynamics We summarise the exciton dynamics from 200 fs − 1 µs timescales in **Figure 3b**. Immediately after excitation, excitons initially populate an unequilibrated (or 'hot') intra-chain charge transfer state (ICT*). Over the first $\sim$ 400 fs, some excitons form interchain species with quantum yield ϕ_{IS}, where ϕ_{IS} depends on the polymer and provides a measure of the density of contact in the polymer microstructure. Excitons that are not transferred to aggregate states thermalise to the ('equilibrated' intra-chain) ICT where they decay to the ground state via radiative and non-radiative pathways (and bimolecularly in the case of higher f_{pump}). In tandem, each interchain species decays directly to the ground state. This may be due to either a limited exciton mobility after the initial 400 fs cooling, or the cascade to lower energy states is made impossible by the occupancy of lower-energy interchain species that are formed early and live longer due to a weaker wavefunction overlap. In either case, after the luminescent IS decay, non-emitting interchain species with longer lifetimes decay on longer timescales until there are no more photoexcited states. At this point, the GSB goes to zero.

The photophysics of TIF-H_2BT is representative of this class of polymer, and is summarised in **Figure 3f**. The transient populations of the various exciton species are simulated from coupled differential equations using the measured values for $\tau_{\mathrm{ICT}^*} = 400$ fs, τ_{ICT} and τ_{IS} for the different polymers. As discussed below, additional non-emissive interchain species with $\tau > \tau_{\mathrm{IS}}$ are required to give a good GSB fit to 1 µs, and the total interchain species formation quantum efficiency (ϕ_{IS}) did not vary substantially with the choice of the distribution of non-emissive IS yields and lifetimes.

Full details of the simulation are given below with tabulated parameters in **Supplementary Tables 4** and **5**. We observe a $\phi_{\text{IS}} \sim 0.048 - 0.075$ varying between the different polymers, except for TIF-H_2BT, which has a higher $\phi_{\text{IS}} = 0.084$. These values are in line with reported ϕ_{IS} determined in a similar way.[26]

Formation of interchain species To determine the quantum yield of interchain species, we use TA spectroscopy. In each of the polymer materials, we track the transient evolution of the GSB after the pump pulse, and use this as a proxy for the total fraction of excited-state species remaining at a given time. In **Supplementary Figure 12**, we show this for each material at two fluences. At the lower fluence in the 1 ns time window, the transient excitation population has approximately bi-exponential kinetics. The shorter lifetime, in all cases where we can independently measure it at exceedingly low fluence, is comparable to (but faster than) the ICT PL decay lifetime (τ_{ICT}). The reason for the shorter τ_{ICT} is second-order recombination involving exciton-exciton annihilation which shortens the lifetime at shorter times. The longer lifetime is due to interchain species.

At the higher fluence, we observe that while most of the population is ICT excitons, these recombine much faster via a mixture of first- and second-order recombination. However, the interchain species recombination rate is largely unaffected by the higher fluence, and the fraction of the population remaining after the ICTs recombine converges between the two fluences. This is shown in **Supplementary Figure 12**. This is because the branching between ICT and interchain species occurs on very early timescales, and the effect of shortening the ICT recombination does little to decrease the interchain CTs remaining after all the ICTs have recombined. Therefore, it follows that at most a very small population of ICTs undergo charge transfer between chains after $\sim$ 400 fs. We speculate that this is owing to the substantially longer lifetime of the interchain species, and a lower density of states.

To investigate the dependency of the decay kinetics on pump wavelength (or E_{pump}), we measure the decay of the transient GSB with different E_{pump}. This is shown in **Supplementary Figure 13**. Between the samples, we set the fluence to match the $\Delta T/T$ at 10 ps, and retrospectively calculate the initial excitation density (n_0) which was $\sim 10^{18}$ cm^{-3}. The calculated fluences and excitation densities summarised in **Supplementary Table 3**. Using this method, we observe that the un-

normalised kinetics are very similar after 10 ps, converging to the same $\Delta T/T$ value by $\sim$ 1 ns, and we have used appoximately constant n_0 for all E_{pump} between samples. However, before 10 ps, we observe a strong dependency of the kinetics on E_{pump}. Pumping higher above the energy gap provides a greater excess energy ($E_{\mathrm{ex.}}$) per excitation, with $E_{\mathrm{ex.}} = E_{\mathrm{pump}} - E_g$. Generally, this leads to a greater degree of GSB cooling on $\sim$ 400 fs timescales, and this is most clear for IDT-H_2BT, whose $n_0 = 2.6 \times 10^{18}$ ± 29 %. In any case, the extraction of the ϕ_{IS} (discussed below) uses data on timescales of $\sim$ 1 ns, where the kinetics converge and are independent of E_{pump}.

Determination of ϕ_{IS} To determine the rate dynamics of the excited states in the polymers, we solved the coupled rate ordinary differential equations (ODEs). Assuming first-order dynamics, the population of state i (n_i) is governed by

$$\dot{n}_j = \sum_i k_{i\to j} n_i - \sum_l k_{j\to l} n_j \tag{8}$$

where $k_{i\to j}$ is the rate of energy transfer from i to j. Using the shorthand that $i = 0$ denotes the ground-state, $i = 1$ denotes the 'hot' ICT state (ICT*), $i = 2$ the 'cooled' and emissive ICT state (ICT), $i = 3$ the highest energy interchain species which may be emissive, later called the 'pinned ICT' (pICT), and $i > 4$ any lower energy interchain charge transfer species which are not emissive (CTs), and assuming down-conversion only, this leads to the coupled equations

$$\dot{n}_1 = -k_{1\to 0} n_1 - \sum_{i>1} k_{1\to i} n_1 \tag{9}$$

$$\dot{n}_2 = -k_{2\to 0} n_2 - \sum_{i>2} k_{2\to i} n_2 + k_{1\to 2} n_1 \tag{10}$$

$$\dot{n}_3 = -k_{3\to 0} n_3 - \sum_{i>3} k_{3\to i} n_3 + k_{1\to 3} n_1 + k_{2\to 3} n_2 \tag{11}$$

$$\dot{n}_{j>3} = -k_{j\to 0} n_j - \sum_{l>j} k_{j\to l} n_j + \sum_{i<j} k_{i\to j} n_i \tag{12}$$

governing the occupation of the states. There is a convenient simplification in our case. If the ICT and interchain species are filled faster than the timescale of the $k_{1\to 2}$ (they are filled before 100 fs), and lower energy states have longer lifetimes (which we observe to be true empirically), then for $t \gg 1/k_{1\to 2}$, $k_{i\to j} = 0$ for $j > i > 1$, and there is no more exciton transfer at later times. This is the condition that intra-chain/interchain branching occurs early.

In this work, we group $k_i = k_{i\to 0} + \sum_{j>i} k_{i\to j}$ (or $k_i = k_{i\to 0}$, assuming fast branching) and write these equations in terms of k_i which is an observable in the case of the ICT*, ICT, and pICT. Additionally, to account for bimolecular recombination of excitons at higher excitation densities (which we require in order to resolve the GSB towards ~ 1 µs), we add a bimolecular recombination term for the ICT ($i = 2$). Finally, while we expect a continuum of possible interchain species due to disorder, each having their own recombination lifetime, we have assumed three discrete populations for $i \geq 2$ with the rate k_i for simplicity. We solve the coupled equations

$$\dot{n}_1 = -k_1 n_1 \tag{13}$$

$$\dot{n}_2 = -k_2 n_2 - k_2^{(2)} n_2^2 + k_{1\to 2} n_1 \tag{14}$$

$$\dot{n}_3 = -k_3 n_3 + k_{1\to 3} n_1 \tag{15}$$

$$\dot{n}_4 = -k_4 n_4 + k_{1\to 4} n_1 \tag{16}$$

$$\dot{n}_5 = -k_5 n_5 + k_{1\to 5} n_1 \tag{17}$$

for the picture summarised diagrammatically in **Supplementary Figure 14**.

By applying the separately observed rates k_1 and k_2 (and k_3 in the case of luminescent pICTs), we fit the remaining parameters to the observed GSB between 1 ps−1 µs in **Supplementary Figure 15**. During the fitting, we note that a strong mutual dependency exists between the pairs of parameters k_i and $k_{1\to i}$, for $i = 3, 4, 5$, and we do not attempt to separate them. Instead, it is more robust to deal with these rates together and we define the quantum efficiency of transfer to a interchain species from $i = 1$ as

$$\phi_{\mathrm{IS}} = \phi_{1\to 3} + \phi_{1\to 4} + \phi_{1\to 5} = \frac{k_{1\to 3} + k_{1\to 4} + k_{1\to 5}}{k_1} \tag{18}$$

in terms of the quantum efficiency of transfer from state i to state j ($\phi_{i\to j}$). The choice of k_i and $k_{1\to i}$ pairs leads to only small variations in the fit ϕ_{IS}. The measured parameters (**Supplementary Table 4**) were fixed during the fitting, and the optimised parameters were summarised in **Supplementary Table 5**.

For all materials in this study, ϕ_{IS} is independent of the pump fluence. While we observe that τ_{ICT} is considerably shortened with increased fluences as exciton-exciton (bimolecular) annihilation occurs, the GSB converges to the same value after SE has finished, as shown in **Supplementary Figure 12** for all our materials. The independence of ϕ_{IS} on fluence indicates that interchain species persisting after ICTs have recombined are formed linearly with excitation density, non-interacting

and likely trapped, and not formed in appreciable yields indirectly from the ICT state, which itself is depleted by bimolecular recombination at high excitation densities. Neither ϕ_{IS} nor τ_{ICT} depend on the wavelength of the pump (**Supplementary Figure 13**).

Determination of $\phi_{\mathbf{PL}}$ In the case that only the ICT is emissive, its ϕ_{PL} is simply given by

$$\phi_{\mathrm{PL}} = \frac{\Phi}{1 - \phi_{\mathrm{IS}}} \tag{19}$$

Where both the ICT and interchain species are luminescent, we must consider the radiative and non-radiative rates of recombination to determine ϕ_{PL} of the ICT and interchain species respectively. In this section, we use the nomenclature: $k_{\mathrm{R},i\to 0}$ as the radiative rate, $k_{\mathrm{NR},i\to 0}$ as the non-radiative rate of recombination from state i, such that $k_{i\to 0} = k_{\mathrm{R},i\to 0} + k_{\mathrm{NR},i\to 0}$. We also define the PLQE of state i as $\phi_{i\to 0} = k_{\mathrm{R},i\to 0}/k_1$. For simplicity, we make the additional approximation that recombination from ICT* is negligible ($k_1 \to \sum_{i\neq 0} k_{1>1}$).

The general equation for the external PLQE (Φ) of such a three emissive-state ($i = 1, 2, 3$) is

$$\Phi = \phi_{1\to 0} + \phi_{1\to 2} \cdot \phi_{2\to 0} + (\phi_{1\to 3} + \phi_{1\to 2} \cdot \phi_{2\to 3}) \cdot \phi_{3\to 0} \tag{20}$$

and in the limit that $\phi_{1\to 0}, \phi_{2\to 3} \to 0$, this equation simplifies to

$$\Phi = (1 - \phi_{1\to 3}) \cdot \phi_{2\to 0} + \phi_{1\to 3} \cdot \phi_{3\to 0} \tag{21}$$

which can be written

$$\frac{\phi_{2\to 0}}{\phi_{3\to 0}} = \left(\frac{\Phi}{\phi_{3\to 0}} - \phi_{1\to 3} \right) \cdot (1 - \phi_{1\to 3})^{-1} \tag{22}$$

The intensity of PL detected $I_i(t)$ from state i at time t is proportional to $\dot{n}_i(t)$ and $k_{\mathrm{R},i\to 0}$

$$I_i(t) \sim k_{\mathrm{R},i\to 0} \cdot \exp(-k_i t) \tag{23}$$

so the time-integrated intensity I_i which we measure is related to

$$I_i \sim \phi_{i\to 0} \cdot n_i(0) \tag{24}$$

In the case that there are two emissive species, $i = 2, 3$, then the ratio of their PL intensities is given by

$$\frac{I_2}{I_3} = \frac{\phi_{2\to0}}{\phi_{3\to0}} \cdot \frac{n_2(0)}{n_3(0)} \tag{25}$$

We can estimate the initial population ratio as the ratio of the formation quantum efficiencies. Although we do not know $\phi_{1\to3}$, we make the substitution $\phi_{1\to3} \to \phi_{\mathrm{IS}}$ (which we calculated earlier with a good degree of certainty) in order to underestimate ϕ_{PL} of the luminescent interchain species. Rearranging gives

$$\frac{\phi_{2\to0}}{\phi_{3\to0}} = \frac{I_2}{I_3} \cdot \frac{\phi_{\mathrm{IS}}}{1-\phi_{\mathrm{IS}}} \tag{26}$$

and combining **Supplementary Equation 19** and **Supplementary Equation 23** gives $\phi_{3\to0}$

$$\phi_{3\to0} = \frac{\Phi}{\phi_{\mathrm{IS}}} \cdot \left(\frac{I_2}{I_3} + 1\right)^{-1} \tag{27}$$

in terms of measured quantities. The calculated ϕ_{PL} the ICT and interchain states are summarised in the main text.

Interplay between ICT and IS luminescence The trend in **Figure 5** is clear, and ϕ_{PL} increases with luminescence energy is clear, as predicted by the energy gap law. We note that the fluorination of the acceptor results (in most cases) in the increase in the ϕ_{PL}, but it also increases the intra-chain ICT energy gap. It is therefore not clear whether a reduced coupling to high-frequency C-H stretch vibrations, thought to be important for exciton quenching,[27, 28] is directly responsible for the increase in ϕ_{PL} or whether the reason is more indirectly the increase of the energy gap upon fluorination. Both mechanisms relate, of course, to different facets of the energy gap law.

Supplementary Note 6: Theoretical calculations

In this paper, we combine molecular mechanics (MM) and molecular dynamics (MD) simulations on a large unit cell (300 Å× 300 Å× 300 Å) containing 24 randomly oriented oligomers ($n = 8$). We apply periodic boundary conditions to build the 3D solid. All MM/MD calculations have been performed with the Materials Studio (MS) 6.0 package using a force-field derived from Dreiding,[29] where torsion potentials between adjacent subunits and between the conjugated cores and the alkyl chains have been benchmarked against density functional theory (DFT) calculations (using the B3LYP functional

and the cc-pvtz basis set).[30–32] The atomic charges have been obtained by fitting the electrostatic potential (ESP charges) calculated at the B3LYP/cc-pvtz level on an isolated dimer.[33] Once the unit cell is built, it is subjected to a 500 ps MD run at high temperature (NVT; $T = 1000$ K) while keeping the density low (~ 0.02 g/cm^3) to favor a random spatial distribution of the oligomers. Then, five successive 500 ps-long MD runs (NPT, $P = 1$ atm) were performed at decreasing temperature (1000 K, 500 K, 400 K, 350 K, 300 K) and, finally, a 2 ns-long MD simulation (NPT; $P = 1$ atm, $T = 300$ K) is performed and snapshots are saved every 5 ps for further analysis.

Supplementary References

[1] Schulz, G. L. *et al.* The PCPDTBT Family: Correlations between Chemical Structure, Polymorphism, and Device Performance. *Macromolecules* **50**, 1402–1414 (2017).

[2] Kim, Y. *et al.* Organic Photovoltaic Devices Based on Blends of Regioregular Poly(3-hexylthiophene) and Poly(9,9-dioctylfluorene-co-benzothiadiazole). *Chem. Mater.* **16**, 4812–4818 (2004).

[3] Sirringhaus, H. *et al.* Two-dimensional charge transport in self-organized, high-mobility conjugated polymers. *Nature* **401**, 685–688 (1999).

[4] Amorim, C. A. *et al.* Determination of carrier mobility in MEH-PPV thin-films by stationary and transient current techniques. *J. Non-Cryst. Solids* **358**, 484–491 (2012).

[5] Lu, L. P. *et al.* The Influence of Side-Chain Position on the Optoelectronic Properties of a Red-Emitting Conjugated Polymer. *Macromol. Chem. Phys.* **214**, 967–974 (2013).

[6] Cao, Y., Parker, I. D., Yu, G., Zhang, C. & Heeger, A. J. Improved quantum efficiency for electroluminescence in semiconducting polymers. *Nature* **397**, 414–417 (1999).

[7] Tanase, C., Blom, P. W. M., de Leeuw, D. M. & Meijer, E. J. Charge carrier density dependence of the hole mobility in poly(p-phenylene vinylene). *phys. stat. sol. (a)* **201**, 1236–1245 (2004).

[8] Chua, L.-L. *et al.* General observation of n-type field-effect behaviour in organic semiconductors. *Nature* **434**, 194–199 (2005).

[9] Yap, B. K., Xia, R., Campoy-Quiles, M., Stavrinou, P. N. & Bradley, D. D. C. Simultaneous optimization of charge-carrier mobility and optical gain in semiconducting polymer films. *Nat. Mater.* **7**, 376–380 (2008).

[10] Bittle, E. G., Basham, J. I., Jackson, T. N., Jurchescu, O. D. & Gundlach, D. J. Mobility overestimation due to gated contacts in organic field-effect transistors. *Nat. Commun.* **7**, 10908 (2016).

[11] Park, K. B. *et al.* High Electrical Performance of Wet-Processed Indium Zinc Oxide Thin-Film Transistors. *IEEE Electron Device Lett.* **31**, 311–313 (2010).

[12] Nikolka, M. *et al.* High operational and environmental stability of high-mobility conjugated polymer field-effect transistors through the use of molecular additives. *Nat. Mater.* **16**, 356–362 (2017).

[13] Thomas, T. *Enhancing Fluorescence and Charge Transport in Disordered Organic Semiconductors.* Doctoral Thesis, University of Cambridge (2018).

[14] Vissenberg, M. C. J. M. & Matters, M. Theory of the field-effect mobility in amorphous organic transistors. *Phys. Rev. B* **57**, 12964–12967 (1998).

[15] Horowitz, G., Hajlaoui, R., Fichou, D. & El Kassmi, A. Gate voltage dependent mobility of oligothiophene field-effect transistors. *J. Appl. Phys.* **85**, 3202–3206 (1999).

[16] Di Pietro, R. *et al.* Coulomb Enhanced Charge Transport in Semicrystalline Polymer Semiconductors. *Adv. Funct. Mater.* **26**, 8011–8022 (2016).

[17] Li, W. *et al.* Mobility-Controlled Performance of Thick Solar Cells Based on Fluorinated Copolymers. *J. Am. Chem. Soc.* **136**, 15566–15576 (2014).

[18] You, J. *et al.* A polymer tandem solar cell with 10.6% power conversion efficiency. *Nat. Commun.* **4**, 1446 (2013).

[19] Clark, J., Silva, C., Friend, R. H. & Spano, F. C. Role of Intermolecular Coupling in the Photophysics of Disordered Organic Semiconductors: Aggregate Emission in Regioregular Polythiophene. *Phys. Rev. Lett.* **98**, 206406 (2007).

[20] Spano, F. C. & Yamagata, H. Vibronic Coupling in J-Aggregates and Beyond: A Direct Means of Determining the Exciton Coherence Length from the Photoluminescence Spectrum. *J. Phys. Chem. B* **115**, 5133–5143 (2011).

[21] Spano, F. C. & Silva, C. H- and J-Aggregate Behavior in Polymeric Semiconductors. *Annu. Rev. Phys. Chem.* **65**, 477–500 (2014).

[22] Mikhnenko, O. V. *et al.* Trap-Limited Exciton Diffusion in Organic Semiconductors. *Adv. Mater.* **26**, 1912–1917 (2014).

[23] Köhler, A., Hoffmann, S. T. & Bässler, H. An Order–Disorder Transition in the Conjugated Polymer MEH-PPV. *J. Am. Chem. Soc.* **134**, 11594–11601 (2012).

[24] Paquin, F. *et al.* Two-dimensional spatial coherence of excitons in semicrystalline polymeric semiconductors: Effect of molecular weight. *Phys. Rev. B* **88**, 155202 (2013).

[25] Dimitrov, S. D. *et al.* Polaron pair mediated triplet generation in polymer/fullerene blends. *Nat. Commun.* **6**, 6501 (2015).

[26] Sheng, C.-X., Tong, M., Singh, S. & Vardeny, Z. V. Experimental determination of the charge/neutral branching ratio in the photoexcitation of π-conjugated polymers by broadband ultrafast spectroscopy. *Phys. Rev. B* **75**, 085206 (2007).

[27] Shafirovich, V. Y., Courtney, S. H., Ya, N. & Geacintov, N. E. Proton-coupled photoinduced electron transfer, deuterium isotope effects, and fluorescence quenching in noncovalent benzo [a] pyrenetetraol-nucleoside complexes in aqueous solutions. *J. Am. Chem. Soc.* **117**, 4920–4929 (1995).

[28] Shizuka, H. & Tobita, S. Proton-induced quenching and hydrogen-deuterium isotope-exchange reactions of methoxynaphthalenes. *J. Am. Chem. Soc.* **104**, 6919–6927 (1982).

[29] Mayo, S. L., Olafson, B. D. & Goddard, W. A. DREIDING: A generic force field for molecular simulations. *J. Phys. Chem.* **94**, 8897–8909 (1990).

[30] Broch, K. *et al.* Measurements of Ambipolar Seebeck Coefficients in High-Mobility Diketopyrrolopyrrole Donor–Acceptor Copolymers. *Adv. Electron. Mater.* **3**, 1700225 (2017).

[31] Lemaur, V. *et al.* On the Supramolecular Packing of High Electron Mobility Naphthalene Diimide Copolymers: The Perfect Registry of Asymmetric Branched Alkyl Side Chains. *Macromolecules* **46**, 8171–8178 (2013).

[32] Olivier, Y. *et al.* 25th Anniversary Article: High-Mobility Hole and Electron Transport Conjugated Polymers: How Structure Defines Function. *Adv. Mater.* **26**, 2119–2136 (2014).

[33] Besler, B. H., Merz, K. M. & Kollman, P. A. Atomic charges derived from semiempirical methods. *J. Comput. Chem.* **11**, 431–439 (1990).
